# Immunophenotypic studies of monoclonal gammopathy of undetermined significance

**DOI:** 10.1186/1472-6890-8-13

**Published:** 2008-11-28

**Authors:** Horatiu Olteanu, Huan-You Wang, Weina Chen, Robert W McKenna, Nitin J Karandikar

**Affiliations:** 1 Department of Pathology, Medical College of Wisconsin, 8701 Watertown Plank Road, Milwaukee, WI 53226, USA; 2 Department of Pathology, UT Southwestern Medical Center, 5323 Harry Hines Blvd, Dallas, TX, USA

## Abstract

**Background:**

Monoclonal gammopathy of undetermined significance (MGUS) is a common plasma cell dyscrasia, comprising the most indolent form of monoclonal gammopathy. However, approximately 25% of MGUS cases ultimately progress to plasma cell myeloma (PCM) or related diseases. It is difficult to predict which subset of patients will transform. In this study, we examined the immunophenotypic differences of plasma cells in MGUS and PCM.

**Methods:**

Bone marrow specimens from 32 MGUS patients and 32 PCM patients were analyzed by 4-color flow cytometry, using cluster analysis of ungated data, for the expression of several markers, including CD10, CD19, CD20, CD38, CD45, CD56 and surface and intracellular immunoglobulin light chains.

**Results:**

All MGUS patients had two subpopulations of plasma cells, one with a "normal" phenotype [CD19(+), CD56(-), CD38(bright +)] and one with an aberrant phenotype [either CD19(-)/CD56(+) or CD19(-)/CD56(-)]. The normal subpopulation ranged from 4.4 to 86% (mean 27%) of total plasma cells. Only 20 of 32 PCM cases showed an identifiable normal subpopulation at significantly lower frequency [range 0–32%, mean 3.3%, p << 0.001]. The plasma cells in PCM were significantly less likely to express CD19 [1/32 (3.1%) vs. 13/29 (45%), p << 0.001] and more likely to express surface immunoglobulin [21/32 (66%) vs. 3/28 (11%), p << 0.001], compared to MGUS. Those expressing CD19 did so at a significantly lower level than in MGUS, with no overlap in mean fluorescence intensities [174 ± 25 vs. 430 ± 34, p << 0.001]. There were no significant differences in CD56 expression [23/32 (72%) vs. 18/29 (62%), p = 0.29], CD45 expression [15/32 (47%) vs. 20/30 (67%), p = 0.10] or CD38 mean fluorescence intensities [6552 ± 451 vs. 6365 ± 420, p = 0.38]. Two of the six MGUS cases (33%) with >90% CD19(-) plasma cells showed progression of disease, whereas none of the cases with >10% CD19(+) plasma cells evolved to PCM.

**Conclusion:**

MGUS cases with potential for disease progression appeared to lack CD19 expression on >90% of their plasma cells, displaying an immunophenotypic profile similar to PCM plasma cells. A higher relative proportion of CD19(+) plasma cells in MGUS may be associated with a lower potential for disease progression.

## Background

Plasma cell dyscrasias show a spectrum of clinical and biological features, ranging from the more indolent forms, such as monoclonal gammopathy of undetermined significance (MGUS) to more aggressive entities viz. plasma cell myeloma (PCM) and plasma cell leukemia. MGUS is the most common, predominantly benign plasma cell disorder and yet a significant number of cases will eventually progress to PCM or related diseases. The overall incidence of progression to myeloma is estimated to be approximately 1% per year [1,2]. However, even with the recent advances in our understanding of the pathogenesis and risk factors in MGUS, it is difficult to predict accurately which subset of patients will transform. It is important to make this distinction because the early identification of patients in the high-risk group would allow the development of effective chemotherapeutic strategies. In general, the majority of MGUS patients have a protracted disease course and die of an unrelated condition, but the potential for progression has been demonstrated even after decades. Thus, chemopreventive regimens would be appropriate in the correct setting [3,4].

A combination of clinical and laboratory parameters is used in distinguishing MGUS form PCM. According to the World Health Organization criteria [5], MGUS is defined by a monoclonal serum protein at less than myeloma levels; fewer than 10% bone marrow plasma cells; the absence of lytic lesions and the lack of myeloma-related organ or tissue impairment. Several biological criteria have been proposed as predictors of the transition of MGUS to PCM or related malignancies, many of them related to the differential expression of plasma cell proteins or molecules that mediate the interaction of plasma cells with the bone marrow environment [6-9]. Flow cytometry is a sensitive and comprehensive technique that has been successfully employed in the diagnosis and follow-up of plasma cell dyscrasias [10-12]. Although immunophenotypic studies have been previously performed in MGUS and PCM patients, primarily for the differential diagnosis of borderline cases, there is relatively little information on the role of routine flow cytometry in identifying MGUS patients with potential for disease progression.

In this study, we examined the immunophenotypic differences in plasma cells in MGUS and PCM and evaluated their utility in predicting disease progression.

## Methods

The University of Texas Southwestern Medical Center Flow Cytometry Laboratory database was searched over a period of ten years for cases with aberrant plasma cell clones, consistent with a plasma cell dyscrasia. Thirty-two patients each were identified as fulfilling the clinical and pathologic criteria for MGUS and PCM, respectively, according to the World Health Organization published guidelines. Their clinical data were obtained through review of medical records. The study was conducted in accordance with the Helsinki Declaration, as approved by the UT Southwestern Institutional Review Board (Protocol # 052008-074).

All patients had a bone marrow sample obtained at the time of the initial presentation immunophenotyped with a "myeloma panel", consisting of antibodies against CD4, CD5, CD8, CD10, CD11b, CD14, CD16, CD19, CD20, CD34, CD38, CD45, CD56, and monoclonal and polyclonal reagents against surface and intracellular kappa and lambda light chains. The following antibody combinations were used (listed in the FITC/PE/PerCP/APC fluorochrome conjugate sequence): CD10/CD19/CD20/CD38, CD14/CD56/CD45/CD38, monoclonal kappa/monoclonal lambda/CD19/CD38, CD5/CD4/CD8/CD19, CD16/CD11b/CD45/CD34, intracellular monoclonal kappa/intracellular monoclonal lambda/CD45/CD38, intracellular polyclonal lambda/intracellular polyclonal kappa/CD45/CD38. None of the patients was receiving therapy related to their condition at the time of the bone marrow biopsy.

For preparation of single cell suspensions for 4-color flow cytometry, bone marrow specimens were incubated for 10 minutes with a standard ammonium chloride lysing solution (1 part sample to 9 parts lysing solution). The samples were then washed twice with phosphate-albumin buffer (PAB), 0.05% sodium azide, and 0.1% bovine serum albumin solution (BSA) and resuspended in RPMI 1640 culture medium supplemented with 5% fetal calf serum. An aliquot of 5 × 10^5 ^cells was stained with a combination of 4 antibodies conjugated with fluorescein isothiocyanate (FITC), phycoerythrin (PE), peridinin chlorophyll protein (PCP), or allophycocyanin (APC) at 2–8°C in the dark for 20 minutes. For the detection of intracellular markers, a permeabilization step was performed prior to the staining procedure. The amount of antibody added was based on the manufacturer's recommendations. At least 30,000 events were acquired routinely using a FACSCalibur flow cytometer with CellQuest software (Becton Dickinson, San Jose, CA). Data analysis was performed using Paint-A-Gate software (Becton Dickinson).

The following antibodies were obtained from Becton Dickinson, unless otherwise specified: CD4 (SK3), CD 5(L17F12), CD8 (SK1), CD10 (W8E7), CD11b (D12), CD14 (MP9), CD16 (NKP15), CD19 (SJ25C1), CD20 (L27), CD38 (HB7), CD45 (2D1), CD56 (MY31), polyclonal kappa and polyclonal lambda (goat IgG; Beckman Coulter, Miami, FL). In general, distinct cell populations were identified using CD45/forward and orthogonal light scatter characteristics, in combination with various antibodies. An antigen was considered positive if >20% of a population cluster expressed the antigen based on a 2% isotypic control threshold.

Statistical analyses of data were carried out in Origin (OriginLab, Northampton, MA) and Excel (Microsoft, Redmond, WA); a p-value < 0.05 was considered statistically significant.

## Results

### Clinical and laboratory characteristics

The MGUS cohort consisted of 16 men and 16 women with a mean age of 59 years (range, 38 to 77 years). The mean age of PCM patients (21 males and 11 females) was 62 years (range, 39 to 84 years). Median follow-up was 80 months for the MGUS group and 36 months for the PCM cohort. Statistically significant differences between the MGUS and PCM group were observed in the hemoglobin concentration, amount of monoclonal component, lactate dehydrogenase, presence of osteolytic bone lesions, number of reduced immunoglobulin classes, and percentage of bone marrow plasma cells estimated by morphologic examination and flow cytometry. In contrast, total serum protein, calcium, and β2-microglobulin did not show significant differences. The patients' pertinent demographic and laboratory data are summarized in Table 1.

**Table 1:** Demographic and laboratory characteristics in patients with MGUS and PCM.

Parameter	MGUS (n = 32)	PCM (n = 32)	*P *value	Reference range
Age	59 ± 12	62 ± 12	0.17	-
M:F	1:1	1.9:1	-	-
Hemoglobin, g/L	121 ± 16	99 ± 21	<0.005	121 – 161
Total protein, g/L	75 ± 13	73 ± 19	0.29	63 – 82
M component, g/L	10 ± 9	38 ± 20	<0.005	-
Calcium, g/L	0.089 ± 0.009	0.09 ± 0.011	0.28	0.084–0.102
β2-microglobulin, mg/L	2.4 ± 1.3	4.9 ± 3.6	0.02	0.7–1.8
Lactate dehydrogenase, U/L	184 ± 50	271 ± 99	<0.005	100 – 190
Osteolytic bone lesions	0%	100%	<0.005	-
Number of reduced Ig classes	0.2 ± 0.6	1.9 ± 0.4	<0.005	-
% plasma cells by morphology	4.2 ± 2.3	41 ± 21	<0.005	-
% plasma cells by flow cytometry	0.7 ± 0.8	19 ± 16	<0.005	-

### FC analysis of plasma cells

**Figure 1:**
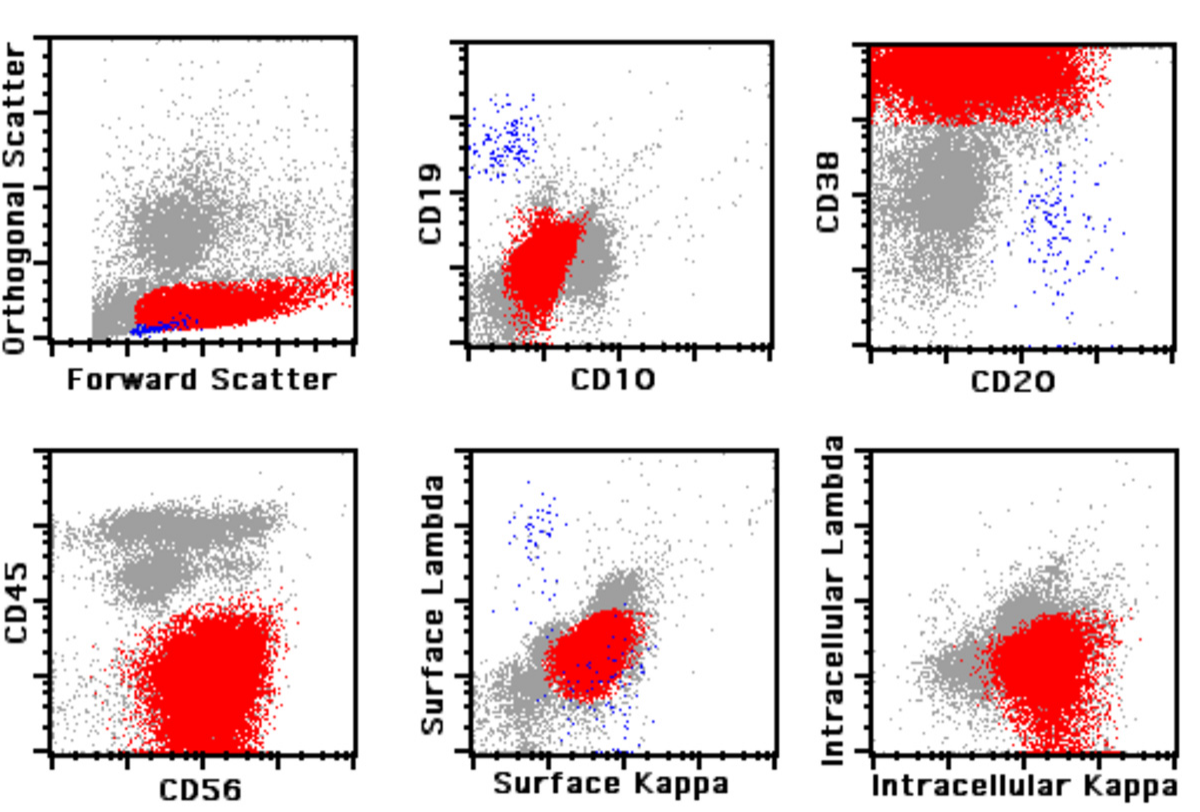
**Flow cytometric analysis of plasma cell myeloma**. Mature B lymphocytes are painted in *blue *and neoplastic plasma cells in *red*.

**Figure 2:**
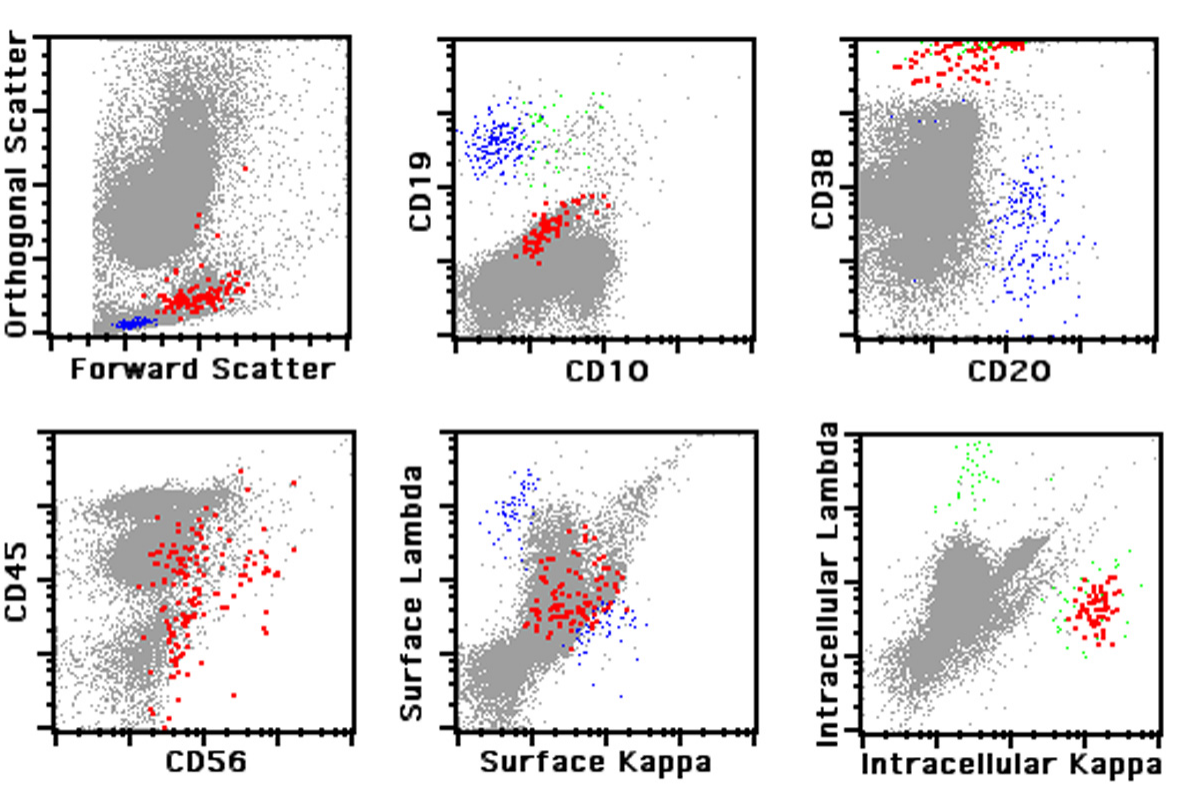
**Immunophenotypic analysis of monoclonal gammopathy of undetermined significance**. Highlighted in *red *are neoplastic plasma cells; mature B lymphocytes are painted in *blue *and polytypic plasma cells in *green*.

Neoplastic plasma cells were defined by bright expression of CD38 and restricted intracellular and/or surface immunoglobulin light chain expression. In order to distinguish normal from aberrant plasma cells, we analyzed the expression of CD19 and CD56 surface antigens. Plasma cells of normal individuals typically express CD19, are negative for CD56 [CD19(+)/CD56(-)] and are polyclonal. Monoclonal plasma cells are either CD19(-)/CD56(+) or CD19(-)/CD56(-) and thus phenotypically aberrant. Figure 1 demonstrates the immunophenotype of a typical case of PCM and Figure 2 illustrates a typical case of MGUS. Of note, neoplastic plasma cells are slightly dimmer for CD38 as compared to residual polytypic plasma cells (Figure 2).

**Figure 3:**
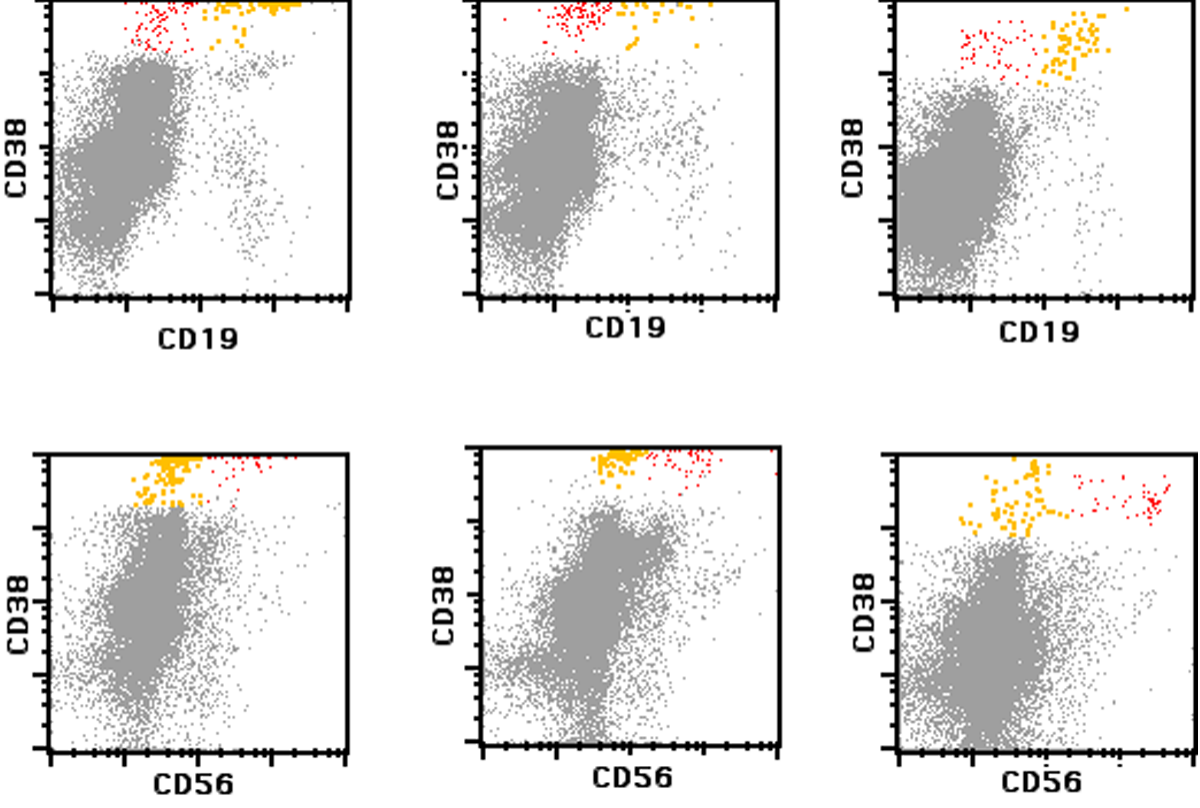
**Comparison of CD19 and CD56 expression in plasma cells of monoclonal gammopathy of undetermined significance (MGUS)**. The *top *row shows CD19 expression in polytypic (*yellow*) and neoplastic (*red*) plasma cells in three different MGUS cases. The corresponding CD56 expression is demonstrated in the *bottom *row.

**Figure 4:**
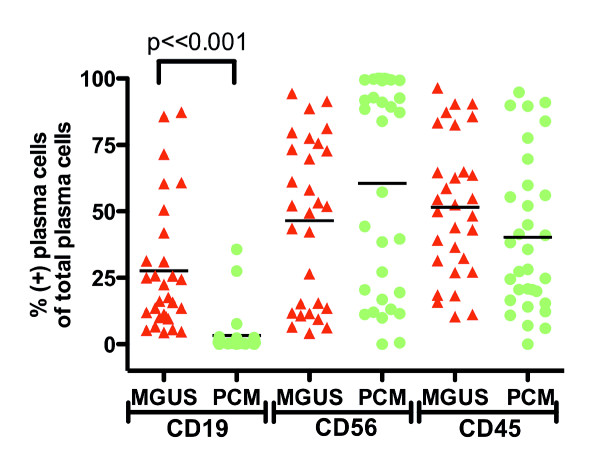
**Comparison of normal plasma cell population size in monoclonal gammopathy of undetermined significance (MGUS) and plasma cell myeloma (PCM)**. MGUS cases (*red*) have a significantly higher CD19(+) plasma cell population as compared to PCM cases (*green*). There are no significant differences in CD56 and CD45 expression. Horizontal bars represent mean values.

All 32 MGUS patients (100%) showed two immunophenotypically different plasma cell populations, one with a normal and the other with an aberrant phenotype (Figure 3). The normal subpopulation accounted for 4.4–86% (mean 27%) of all bone marrow plasma cells (Figure 4). In contrast, only 20 of 32 PCM cases (62.5%, p < 0.001) showed a phenotypically normal population, ranging from 0–32% (mean 3.3%); this population was significantly smaller than in the MGUS group (Figure 4). This finding reflects the most striking difference between the two patient cohorts, which was CD19 expression of total plasma cells.

Total plasma cells in MGUS were more likely to express CD19 (13/29 cases, 45%) than those in PCM (1/32 cases, 3.1%, p < 0.001). However, the opposite was true for surface immunoglobulin light chain expression, were the majority of PCM cases had surface immunoglobulins (21/32, 66%) as compared to MGUS (3/28, 11%, p < 0.001). The frequency of CD56(+) total plasma cells was similar in MGUS (23/32, 72%) and PCM (18/29, 62%, p = 0.29). There were also no significant differences in CD45 expression (20/30, 67% in MGUS vs. 15/32, 47% in PCM, p = 0.1). A comparison of antigen expression in plasma cells of MGUS vs. PCM is summarized in Figure 4.

The expression of CD19 in MGUS plasma cells was significantly brighter than in PCM: the average ± standard deviation (SD) mean fluorescence intensity (MFI) of CD19 in MGUS was 430 ± 34, whereas the average ± SD MFI in PCM 174 ± 25 (p << 0.001). Although PCM plasma cells expressed CD38 at a dimmer level as compared to MGUS, there was no statistically significant difference in CD38 MFI (7020 ± 451 in MGUS vs. 6365 ± 420 in PCM, p = 0.130).

### MGUS patients with progression of disease

Two out of 6 (2/6, 33%) MGUS cases with >90% CD19(-) plasma cells showed progression of disease, whereas none of the 26 (0/26, 0%) cases with <10% CD19(-) plasma cells evolved to PCM. Progression was established according to the criteria defined by the International Myeloma Working Group [13]. Table 2 summarizes laboratory data of the two MGUS patients with progression of disease. With the exception of the percentage of bone marrow plasma cells and the amount of the urinary monoclonal protein, which are presented as a range from diagnosis to the latest follow-up, the rest of the laboratory data are from the latest follow-up. Both patients had an increase in the percentage of bone marrow plasma cells and Bence-Jones proteinuria within a similar interval (time from diagnosis to progression = 48 months, each). As a group, the MGUS patients had an estimated progression-free survival (PFS) of 67% at 5 years, while the cases with <10% CD19(-) plasma cells had an estimated PFS of 100% at 5 years. At the time of completion of this study, the two MGUS patients which showed related organ or tissue impairment (ROTI), specifically renal insufficiency, had not received myeloma-directed chemotherapy, but were followed and treated for their renal impairment.

**Table 2:** Laboratory characteristics of MGUS patients with progression of disease.

Parameter	MGUS #1	MGUS #2	Reference range
Hemoglobin, g/L	133	140	121 – 161
Total protein, g/L	74	45	63 – 82
Creatinine, g/L	0.019	0.046	0.006–0.012
Calcium, g/L	0.089	0.091	0.084–0.102
β2-microglobulin, mg/L	4.9	12.1	0.7–1.8
LDH, U/L	204	223	140–271
% PC by morphology	4.6 ⇒ 10	8.5 ⇒ 12	-
Urinary M component (mg/24 hrs)	647 ⇒ 1,640	755 ⇒ 1,786	-
κ:λ light chain ratio	79.4	44.8	0.26–1.65

## Discussion

The distinction between MGUS and PCM is an important one for diagnostic, therapeutic, and prognostic purposes. Furthermore, the ability to identify MGUS patients with potential of progression of disease can offer to this group of patients early treatment options without increasing the morbidity of what is otherwise a relatively indolent entity. Flow cytometry has been proven as an effective tool in distinguishing normal plasma cells from their neoplastic counterparts by using both three-color [10,14,15] and four-color staining [11]. This method is useful not only in separating MGUS from PCM cases [10,14,15], but also in evaluating minimal residual disease and offering predictive information on therapeutic outcomes [12,16,17].

We undertook the present study to evaluate the efficacy of our flow cytometry antibody cocktails in detecting plasma cells in MGUS and PCM. Based on the immunophenotypic profiles obtained from this analysis, we then extended our study to investigate the clinical utility of some routinely used surface and intracellular markers in predicting MGUS cases at risk for progression. Our study demonstrates that bone marrow specimens from all MGUS cases had two distinct subpopulations of plasma cells, according to their expression of CD19 and CD56. One of these populations is found in all cases of MGUS and is considered phenotypically normal and polyclonal, while the second one is monoclonal and phenotypically aberrant. In contrast, PCM patients had a normal subpopulation in only less than two thirds of the cases, and these plasma cells accounted for an average of less than 4% of the total bone marrow plasma cells. The findings are similar to those reported in previous studies that employed three-color techniques [10,14,15]. The percentage of residual normal plasma cells is thus an effective parameter in the differential diagnosis of PCM vs. MGUS.

There are numerous studies that have examined the immunophenotypic characteristics of plasma cells in PCM [18,19] and/or MGUS [10,14,15,20,21]. Some of these authors have used CD138 as a selective marker for separating immunophenotypically normal from neoplastic plasma cells, and a recent report of the European Myeloma Network has demonstrated that a combination of CD138, CD38 and CD45 antibodies allows the most reproducible detection of neoplastic plasma cells [22]. Others have employed a CD38/CD45/CD19/CD56 antibody cocktail tube to distinguish neoplastic plasma cells from their normal counterpart [11]. By selecting plasma cells using a similar approach and in combination with light chain restriction (5 ≤ κ/λ ratio ≤ 0.5), we felt we can discriminate between monoclonal and polyclonal plasma cells present in the same specimen and separate them from other hematopoietic cells, in particular hematogones. Furthermore, the frequency of CD19 and CD56 expression in PCM plasma cells detected in the present study is similar to the range reported in the literature, namely, <1–10% and 71–79%, respectively. Interestingly, the present study found a higher frequency of CD45 expression than in the large series reported by Lin and colleagues (45% in our cases vs. 9% reported by Lin et al.)[19]. However, this value is similar to that reported in a series of previously characterized PCM cases in our laboratory [23]. A range of CD45 expression has been demonstrated in other studies [24,25] and may be due to genetic and biologic heterogeneity of neoplastic cells in each study [25,26].

In the present study, the number of MGUS patients lacking CD19 expression in >90% of their total bone marrow plasma cells was significantly lower than in the PCM group. One third of MGUS patients with a predominance of CD19(-) plasma cells had evidence of progression of disease, whereas none of those with <10% of CD19(-) total plasma cells did. This indicates that the ratio of immunophenotypically abnormal to total bone marrow plasma cells is helpful not only in providing a flow cytometric separation of PCM from MGUS, but may also have predictive information when evaluating MGUS patients with potential for disease progression. While somewhat limited by the length of follow-up and the number of cases analyzed, our findings are consistent with the results of a recent study, which evaluated a large cohort of MGUS and smoldering myeloma patients and found that 24% of MGUS patients with >95% aberrant plasma cells of total bone marrow plasma cells progressed to PCM, as compared to only 4% of cases with <95% of aberrant plasma cells [27]. Of note, our cut-off used for defining MGUS with potential for disease progression (<10%) was not identical to the one in the afore-mentioned study (<5%) which may pose an issue in terms of reaching a consensus and would be also important in the setting of minimal residual disease monitoring. Apart from the latter study, there are few reports in the literature addressing the utility of flow cytometric markers used in the routine practice that arrived to a similar conclusion [28]. In contrast, there is extensive information on the immunophenotypic profile of plasma cells associated with an active of aggressive disease course. Some of these markers, such as CD86, CD126, CD130, HLA-1, β2-microglobulin, MPC-1, CD49e, CD200 [7-9] may be less seldom employed in routine flow cytometry panels. Other studies used microarrays to evaluate global gene expression for determining the molecular fingerprint associated with malignancy [6,29-32]. These authors were able to identify genes that are involved in the sequential transformation of normal to malignant plasma cells and thus can be useful in identifying the progression of MGUS to PCM.

These and other reports underscore the existence of a biological continuum from normal plasma cells, to MGUS, and to PCM or other related malignancies. MGUS patients are largely asymptomatic and are progressing over time to more aggressive disease at a relatively low rate (1% per year), which does not diminish over time [1,2]. Genetic instability and bone marrow microenvironmental changes are some of the factors that have been postulated to play a role in progression. For prognostic purposes, a risk-stratification system has been proposed, based on three risk factors: amount of the serum monoclonal component, immunoglobulin type and serum free light chain ratio [33]. In addition, percentage of aberrant bone marrow plasma cells and DNA aneuploidy were strong predictors for progression-free survival in another recent study [27].

## Conclusion

In summary, we evaluated the usefulness of various routine immunophenotypic markers in characterizing cases of MGUS and in a small series, identified patients lacking CD19 on the majority of their bone marrow plasma cells as being more likely to show progression of disease. Based on similar findings reported in a comprehensive study [27], it appears that flow cytometric evaluation of CD19 expression in bone marrow plasma cells could be incorporated as an adjunctive test in the current patient stratification schemes.

## Competing interests

The authors declare that they have no competing interests.

## Authors' contributions

HO analyzed the data, carried out statistical analysis and wrote the manuscript. NJK generated the idea for the study and supervised the study. HYW, WC and RWM provided intellectual input and critically revised the manuscript. All authors read and approved the final manuscript.

## Pre-publication history

The pre-publication history for this paper can be accessed here:


